# Fast Growth at a Physiological Cost: Comparing *Odontobutis potamophilus* in Recirculating Aquaculture and Rice–Crayfish–Fish Culture

**DOI:** 10.3390/biology15191706

**Published:** 2026-09-24

**Authors:** Shasha Zhao, Haoran Liu, Jikai Ding, Kun Xiao, Jiahong Shi, Xin Zhang, Ladeertu A, Yang Li, Xinye Wu, Yongjin Li, Liwen Wang, Guofu Huang, Lei Zhang

**Affiliations:** 1 School of Chemical Engineering and Environment, Weifang University of Science and Technology, Weifang 262700, China; zhaoshasha1131@126.com (S.Z.); hgflh007@163.com (G.H.); 2 College of Life Science, Huzhou University, Huzhou 313000, China; adjkemail@163.com (J.D.); a842041019@163.com (J.S.); 02572@zjhu.edu.cn (Y.L.); wxyray1108@163.com (X.W.); liyongjin@huznu.edu.cn (Y.L.); wanglw0301@163.com (L.W.); 3 Institute of Hydrobiology, Chinese Academy of Sciences, Wuhan 430072, China; xiaokun@ihb.ac.cn; 4 College of Forestry and Biotechnology, Zhejiang A&F University, Hangzhou 311300, China; 5 Fisheries Service Center of Urat Front Banner, Bayannur 014400, China; zhx731103@163.com (X.Z.); ladeertua@126.com (L.A.); 6 Huai’an Research Center of Hydrobiology, Huai’an 223002, China

**Keywords:** *Odontobutis potamophilus*, culture model, growth parameters, feeding strategies, serum biochemical parameters

## Abstract

The freshwater sleeper (*Odontobutis potamophilus*) is a high-value freshwater fish endemic to China, farmed mainly in rice–crayfish–*Odontobutis potamophilus* integrated culture (RCO) and recirculating aquaculture systems (RAS). How these models differ in growth, physiological status, and feeding remains unclear. The same batch of fry was reared for three months (92 days) in six paddy fields (RCO, 3 individuals/m^2^, no artificial feed, with access to natural food organisms and stocked crayfish) and six RAS tanks (6 individuals/m^2^, fed *Cirrhinus molitorella* and pellet feed twice daily). Growth indicators were calculated from individual data of the sampled fish (*n* = 138 per model). Per group, 150 fish were sampled for stomach contents, 138 for morphometrics, and six for serum alanine aminotransferase (ALT), glutamic–oxaloacetic transaminase (AST), and glucose (GLU) assays; muscle nutritional composition was also determined. Relative weight gain rate (RWG) and specific growth rate in wet weight (SGRw) were significantly higher in RAS, with positive allometric growth in both models. Muscle crude protein was higher in RCO, whereas crude fat and serum ALT, AST, and GLU levels were higher in RAS, suggesting a heavier metabolic burden on the liver in RAS, which awaits confirmation with a broader panel of liver function markers. RCO stomachs contained nine food categories but had a 38% vacuity rate, whereas RAS fish consumed only *C. molitorella* (86% occurrence, 14% vacuity). Because stocking density and feeding regimen differed simultaneously between the two models, the observed differences reflect the overall effects of each culture model rather than the effect of the rearing system alone. These findings reveal a growth-metabolism trade-off between the two culture models as currently practiced: RAS produced faster growth accompanied by higher serum transaminase levels and body fat, whereas RCO maintained lower metabolic markers with slower growth. Moderate supplementary feeding in RCO and dietary diversification in RAS may reconcile yield and fish health.

## Simple Summary

Freshwater sleeper is a small, high-value freshwater fish farmed mainly in China, where it is raised under two very different systems: indoor tanks that continuously recycle water and rice paddies where fish live together with rice plants and crayfish. Farmers therefore face a practical question: which system produces healthy fish more efficiently? To answer this, we raised the same batch of young fish under both systems for three months and then compared their growth, body composition, blood chemistry, and stomach contents. Fish in the recycling tanks, where food was always abundant and easy to obtain, grew much faster. However, these fish accumulated more body fat and showed blood signs of an overburdened liver. Fish in the rice paddies grew more slowly because their natural food was scattered and sometimes hard to find, but they stayed lean, rich in protein, and physiologically stable. Our results reveal a clear trade-off between fast growth and good health. We therefore suggest that paddy farmers provide modest extra feed, while tank farmers diversify the diet and introduce water flow so that both systems can produce more fish without compromising fish health.

## 1. Introduction

The freshwater sleeper (*Odontobutis potamophilus*) is a premium freshwater fish species in China [1]. Over the past decade, wild populations have continued to decline due to overfishing and habitat fragmentation [2]. Against this backdrop, the industry primarily relies on two culture models to ease the market supply–demand imbalance. These are rice–crayfish–*Odontobutis potamophilus* integrated culture (RCO) and recirculating aquaculture system (RAS) [3,4]. RAS represents a highly intensive production paradigm [5]. It has gained rapid worldwide adoption in the aquaculture industry [6]. This culture model seeks higher specific growth rates and better feed conversion ratios. Such adoption is driven by technical advantages in water conservation, high efficiency, and low emissions. However, recent studies have reported that fish reared in culture models oriented toward rapid growth exhibit elevated stress indicators and liver injury markers [7,8]. In the rice–crayfish–fish integrated culture, rice plants provide shade, the water is relatively shallow, and the system supports a rich diversity of natural food organisms at typically low stocking densities [9]. Its diverse supply of natural food may also significantly alter fish feeding strategies and dietary composition [10,11]. Unlike the sufficient feed supply in RAS, natural food organisms in RCO are spatiotemporally dispersed, requiring fish to engage in frequent foraging and cruising activities. Such sustained activity may exert a positive effect on skeletal and muscular development [12,13]. Whether such activity ultimately translates into improved growth performance and physiological status under actual production conditions remains an open question.

Growth and feeding are core indicators for evaluating fish growth performance and physiological status. Growth differences are reflected in final body weight and standard length. More fundamentally, they arise from differences in feeding adaptability to the food composition of the culture environment and in energy acquisition strategies [14,15]. Water temperature, food availability, and foraging ability are core factors that shape individual growth trajectories by regulating metabolic rate and energy conversion efficiency [16]. While optimal temperatures and sufficient food resources accelerate growth, frequent disturbances or elevated foraging pressure suppress weight gain instead [17]. Under RAS, a consistent, stable artificial diet paired with a feeding regime directs the majority of fish energy allocation towards somatic growth. In contrast, RCO presents a diverse but spatiotemporally dispersed natural food spectrum, which requires fish to allocate more energy to searching, selection, and predation [14,18]. This difference in food environments may drive the divergence of feeding efficiency and growth performance between the two models. A systematic comparison of these differences can provide a theoretical basis for optimizing feeding strategies and culture models for *O. potamophilus*.

Accordingly, the present study utilized the same batch of *O. potamophilus* fry, which were cultured for three months (92 days) under two culture modes: RAS and RCO. To clarify the growth differences between the two culture modes and analyze the overall effects of the two culture modes, which differ simultaneously in rearing system, stocking density, and feeding regimen, on the growth strategy and physiological status of *O. potamophilus*, this study systematically measured growth performance, body nutrient composition, serum biochemical parameters, and feeding-related indicators. The obtained results can provide a theoretical basis for optimizing the two culture modes and facilitate the simultaneous improvement of culture yield and fish health in *O. potamophilus* farming.

## 2. Materials and Methods

### 2.1. Experimental Fish and Rearing Conditions

The experimental *O. potamophilus* were purchased from Wuhan Deshen Farmers’ Professional Cooperative. The fry were randomly divided into two groups and reared for three months (92 days) under two culture models (i.e., RCO and RAS). Randomization was performed at the experimental-unit level: the fish were randomly allocated to six independent experimental units per model, namely six paddy fields for RCO (2000 individuals per field) and six tanks for RAS (1200 individuals per tank). Each paddy field or tank served as one independent experimental unit. The fry were transported to the two experimental facilities via oxygenated live fish transport vehicles, with water temperature maintained consistent with that of the source water throughout the transportation process. Upon arrival, the fish were held in temporary acclimation tanks at each facility for two weeks. Water temperature was gradually adjusted from the transport temperature to the rearing temperature of the respective culture model (25–26 °C) over the acclimation period. During acclimation, fish were fed small daily rations of a commercial feed (Shaoxing Damuzhi Aquarium Products Co., Ltd., Shaoxing, Zhejiang Province, China). Feeding behavior was monitored daily. Fish were transferred to the experimental systems after they resumed normal feeding and showed no signs of stress. At the initiation of the rearing trial, the RCO group had an initial body weight of 0.87 ± 0.08 g, while the RAS group had an initial body weight of 0.91 ± 0.09 g; for initial standard length, the RCO group was 1.74 ± 0.15 cm, and the RAS group was 1.79 ± 0.18 cm. No significant intergroup differences were detected for either trait.

The RCO experimental site is located at Wuhan Xiaoyao Agricultural Technology Development Co., Ltd., in Leiling Village, Jiangxia District, Wuhan City, Hubei Province. The rice variety planted in the experimental paddy field was Nangeng 46. The measured water quality parameters were as follows: water temperature was 26.8 ± 2.4 °C (mean of daily measurements), dissolved oxygen content was 6.74 ± 0.42 mg/L, ammonia nitrogen content was less than 0.1 mg/L, and pH was 7.04 ± 0.52. In the RCO system, no supplementary artificial feed was provided during the experiment; fish had access to naturally occurring food resources in the paddy field ecosystem, including plankton and benthic invertebrates, as well as stocked crayfish. The RAS experimental site is situated at the Wuhan Zhenyan Agricultural Products Professional Cooperative in Chenggong Modern Urban Agricultural Development Zone, Caidian District, Wuhan City, Hubei Province. The RAS facility maintained the following water quality parameters: water temperature 25.3 ± 1.1 °C, dissolved oxygen 6.79 ± 0.41 mg/L, ammonia nitrogen below 0.1 mg/L, and pH 7.02 ± 0.56. The water recirculation frequency was one cycle per hour, and the tangential flow velocity was maintained at 0.1–0.3 m/s. In the RAS system, fish were fed *Cirrhinus molitorella* as the main food source, supplemented with fish paste and high-protein pellet feed twice daily (at 8:00 and 17:00). At each feeding, feed was provided to apparent satiation, and no uneaten feed was observed within one hour after feeding. The nutritional composition of the artificial feed was as follows: crude protein not less than 45.0%, crude fat not less than 5.0%, and moisture not more than 12.5%. Both facilities followed standardized management protocols appropriate to their respective culture models. The stocking densities of the two models followed the routine commercial practice of each system, reflecting actual production conditions rather than an experimental gradient. Rearing parameters of *O. potamophilus* under both culture models are shown in Table 1. No chemical fertilizers or pesticides were used throughout the experiment.

At the end of the experiment, all fish in each paddy field and each tank were harvested by complete drainage. Subsequently, 150 healthy *O. potamophilus* were randomly collected from the two culture sites. In the RCO system, fish were collected from three locations within each ring ditch (both ends and the midpoint) and two diagonal locations within each paddy field. In the RAS system, fish were collected from the four corners and the center of each tank. This multi-point sampling strategy was employed to minimize selection bias and ensure representativeness. The 150 individuals per system (25 from each experimental unit) were collected from multiple points within each unit to represent the whole population of each model. They were used for stomach content analysis, size–frequency description, and the individual-level calculation of growth indicators. A sample size of 150 individuals per system is consistent with standard practice in aquaculture growth and feeding studies [11]. They were rapidly anesthetized with MS-222 (100 mg/L; Sigma-Aldrich, St. Louis, MO, USA). Then they were placed on ice for subsequent measurement of growth and feeding habits.

### 2.2. Sample Collection and Processing

The collected *O. potamophilus* individuals were assessed for external physical condition. Individuals with intact body surfaces and normal postural status were immediately anesthetized, after which morphological measurements were conducted. Among the 150 individuals collected from each system, 12 individuals in the RCO system exhibited external body damage (scale loss or fin injury) or abnormal body posture incurred during sampling and transport. To ensure comparability between the two culture models, 138 individuals were retained for morphological measurements in each system. In the RCO system, the 12 damaged individuals were excluded, and 138 intact individuals were used. In the RAS system, 138 individuals were randomly selected from the 150 collected to match the sample size of the RCO system. In total, 138 individuals from each culture model were measured. Standard length (SL, accurate to 0.01 cm) was measured with vernier calipers, and mouth width (MW, accurate to 0.01 cm) was additionally recorded. Body weight (BW, accurate to 0.01 g) was quantified via electronic balance. These data were used to fit the length–weight relationship and the length–mouth gape width relationship.

Dietary analysis requires dissecting specimens to retrieve gastric contents, and this procedure is independent of the external morphological characteristics of the study subjects. The present analysis included a total of 150 individuals. As only 138 individuals had complete data for standard length, body weight, and mouth gape width, the subsequent analyses of the length–weight relationship, the mouth gape width–length relationship, and the standard length–prey size relationship were all based on this sample size. The fish were euthanized, and their entire digestive tracts were completely isolated from the esophagus to the pyloric caeca. Both ends of each isolated digestive tract were ligated with fine sutures, after which the samples were fixed in 4% neutral formaldehyde solution for subsequent stomach content identification. Food composition in the two culture models was compared with feeding habits reported for pond culture (PC) and rice field culture (RFC) in the literature [11].

During laboratory identification of stomach contents, the food type with the highest mass proportion was recorded for each fish as its primary food source. This dominant-item approach was adopted to characterize the main energy source of each individual, and the occurrence data across 150 individuals per model were used to describe the dietary structure at the population level. If stomach contents were highly degraded and chyme-like, or no visible food was present, the stomach was recorded as empty. Intact or lightly digested prey items were directly identified to species level by morphology. If intact *C. molitorella* individuals were found, their standard length and body depth were measured (to 0.01 cm). Moderately digested samples were identified by hard tissue characteristics such as pharyngeal teeth and scale rows. Ten *O. potamophilus* with relatively intact *C. molitorella* in their stomachs were selected from each culture model (20 individuals in total). The standard length and body depth of the *C. molitorella* in their stomachs were measured. The relationship between these two measurements was then analyzed.

Among 138 individuals with intact external morphology, six *O. potamophilus* individuals per experimental system were randomly selected for blood biochemical analysis. Following anesthesia, blood was collected from the caudal vein, with a sampling volume of 2 mL per fish. For each model, one fish was randomly selected from each of the six experimental units (six paddy fields for RCO and six tanks for RAS), so that each fish represented one independent experimental unit (*n* = 6 per model). Blood was collected into tubes without anticoagulant. After clot formation at 4 °C for 12 h, the samples were centrifuged at 4000 rpm for 10 min, and the supernatant was collected as serum. Blood collection was performed in the morning, before the first daily feeding in the RAS. The serum was stored at −80 °C and sent to Hefei Laier Biotechnology Co., Ltd., Hefei, China. Three indices were measured. These were alanine aminotransferase (ALT), glutamic–oxaloacetic transaminase (AST), and glucose (GLU). The body’s nutritional composition was determined following the national standard method, with four parameters measured: crude protein, ash, crude fat, and moisture. Moisture, crude protein, and ash were expressed on a wet-weight basis, whereas crude fat was determined on dried samples and expressed on a dry-matter basis.

### 2.3. Data Analysis

Relative weight gain rate (RWG, %) was used to calculate the relative weight gain of *O. potamophilus* during the experiment. The formula is as follows:

RWG = (W_t_ − W_0_)/W_0_ × 100

Here, W_0_ and W_t_ are the initial and final body weights of *O. potamophilus* (g), respectively.

Specific growth rate in wet weight (SGR_w_, %) was used to evaluate the specific growth in wet weight of *O. potamophilus*. The formula is as follows:

SGR_w_ = (lnW_t_ − lnW_0_)/t × 100

Here, t is the duration of the experiment (days). SGR_w_ was calculated for each individual fish using the mean initial body weight of the respective group and the final individual body weight and then averaged (*n* = 138 per model; t = 92 days).

Fulton’s condition factor (K) was used to evaluate the body condition of *O. potamophilus*. The formula is as follows:

K = 100 × BW/SL^3^

Here, BW is the body weight (g), and SL is the standard length (cm) of *O. potamophilus*.

Occurrence rate (OR, %) was used to evaluate the frequency of specific food items in the digestive tract of *O. potamophilus*. The formula is as follows:

OR = N_m_/N × 100

Here, N_m_ is the number of dissected *O. potamophilus* in which a certain food type was recorded as the dominant stomach content. N is the total number of dissected *O. potamophilus*.

### 2.4. Statistical Analysis

Excel and Origin Pro 8.0 were used for data processing and correlation analysis. Different models were compared by their R-squared values. The model with the highest R-squared value was selected as the optimal model. SPSS 26.0 was used for statistical analysis. For growth performance, all indicators were calculated from individual data of the sampled fish (*n* = 138 per model, 25 individuals from each of the six experimental units, collected from five points within each unit to maximize representativeness). Morphological measurements and regression analyses were also performed at the individual level. For blood biochemical indices, one fish was randomly selected from each experimental unit (*n* = 6 per model). All data were first tested for normality using the Shapiro–Wilk test and for homogeneity of variances using Levene’s test. Differences between the two culture models were compared using independent-samples Student’s *t*-tests. Data that still did not follow a normal distribution after transformation were analyzed using the Mann–Whitney U test. Correlations between predator and prey size were evaluated using Pearson’s correlation analysis. *p* < 0.05 was set as the significance level. Graphs were plotted using GraphPad Prism 8.

## 3. Results

### 3.1. Comparison of Growth Characteristics

The initial standard length (ISL) of *O. potamophilus* was 1.74 ± 0.15 cm in the RCO group and 1.79 ± 0.18 cm in the RAS group. The initial body weight (IBW) of *O. potamophilus* was 0.87 ± 0.08 g in the RCO group and 0.91 ± 0.09 g in the RAS group. No significant differences were detected in initial body weight or initial standard length (ISL) between the two groups. After 92 days of culture, RAS was significantly higher than RCO across five growth performance indicators: final standard length (FSL), final body weight (FBW), body weight gain (BWG), relative weight gain rate (RWG), and specific growth rate in wet weight (SGR_w_) (Table 2). Fulton’s condition factor was significantly higher in RAS than in RCO (2.15 ± 0.36 vs. 1.77 ± 0.23, *p* < 0.001).

In this study, *O. potamophilus* in RCO showed a standard length distribution of 6.12–12.91 cm. The mean standard length was 8.13 ± 1.82 cm. The body weight distribution was 3.25–43.28 g. The mean body weight was 11.26 ± 8.73 g. However, these indices were higher in RAS, with a standard length distribution of 6.24–13.06 cm (mean 8.81 ± 1.65 cm) and a body weight distribution of 3.84–55.4 g (mean 17.04 ± 11.53 g). The relative frequency distribution of standard length showed that individuals with standard lengths between 6 and 9 cm accounted for more than 60% of the total sample in both culture models. In RCO, the 6–7 cm group had the highest relative proportion at 37% (Figure 1A). In RAS, the 7–8 cm group had the highest relative proportion at 29% (Figure 1B). The occurrence frequency of large standard length groups (above 11 cm) was low in both culture models. The relative frequency distribution of body weight showed that individuals with body weight within 20 g accounted for more than 70% of the total sample in both culture models. The occurrence ratio of high body weight groups (>20 g) was low. In RCO, the 0–10 g group had the highest relative proportion at 56.5% (Figure 1C). In RAS, the 10–20 g group had the highest relative proportion at 36.2% (Figure 1D).

The length–weight relationship of *O. potamophilus* exhibited positive allometric growth under both culture modes, with the power exponent exceeding 3 in both treatments. In RCO, the allometric growth equation was BW = 0.0114 × SL^3.21^ (*n* = 138; R^2^ = 0.97) (Figure 2A). In RAS, the allometric growth equation was BW = 0.0068 × SL^3.53^ (*n* = 138; R^2^ = 0.95) (Figure 2B). The exponent b was significantly greater than 3 in both models (RCO: b = 3.21 ± 0.05, t = 4.09, *p* < 0.001; RAS: b = 3.53 ± 0.07, t = 7.35, *p* < 0.001), confirming positive allometric growth. The exponent b differed significantly between the two models (t = −3.67, *p* < 0.001). Standard length and body weight showed a highly significant power function relationship in both culture models, with good model fit and a higher power exponent in RAS.

### 3.2. Food Composition Differences

The occurrence frequencies of food items of *O. potamophilus* under different culture models are presented in Figure 3, and the number of food occurrences is shown in Table 3. The food composition in RCO exhibited high taxonomic diversity, with nine distinct food categories identified. *C. molitorella* constituted the dominant food source in the diet, being recorded as the dominant food item in 46% of the stomachs. Additional natural prey items, including *Pseudorasbora parva* and gobies, were also detected in the diet. However, the vacuity rate of *O. potamophilus* in this culture model reached 38%. Only one prey item, *C. molitorella*, was detected in the stomach contents of fish from the RAS, recorded as the dominant food item in 86% of the stomachs. No additional prey items were observed, and the stomach vacuity index was only 14%. In pond culture and rice field culture, crayfish constituted the primary food component, with occurrence rates of 82.67% and 60.67% and recorded food category counts of 8 and 11, respectively. Overall, the food structure in RCO differs from that observed in traditional rice field culture. The RAS exhibits a uniform and stable feeding regime, characterized by high food availability but low trophic diversity. Differences in food structure among the four culture models showed that food availability and diversity jointly influenced the feeding of *O. potamophilus*.

Mouth width (MW) of *O. potamophilus* ranged from 1.13 to 2.68 cm in RCO (mean 1.62 ± 0.36 cm) and 1.14 to 2.69 cm in RAS (mean 1.73 ± 0.35 cm). The mean MW was significantly higher in RAS than in RCO (independent-samples *t*-test, t = 2.63, *p* < 0.05). The relationships between standard length and MW in the two culture models were MW = 0.197 × SL + 0.0215 (*n* = 138; R^2^ = 0.97) (Figure 4A) and MW = 0.2066 × SL − 0.083 (*n* = 138; R^2^ = 0.95) (Figure 4B). The slopes of the two regressions did not differ significantly between the two models (ANCOVA, interaction term: t = −1.94, *p* = 0.054), indicating that MW increased proportionally with standard length in both models. Standard length and mouth width showed a highly significant linear relationship, with good model fit.

In RCO, the standard length of relatively intact *C. molitorella* in the stomachs of *O. potamophilus* ranged from 0.62 to 5.66 cm. Body depth ranged from 0.13 to 1.24 cm. The relationship between SL and PSL was PSL = 0.905 × SL − 4.6056 (*n* = 10; R^2^ = 0.86) (Figure 5A). The relationship between MW and BD was BD = 0.958 × MW − 0.8813 (*n* = 10; R^2^ = 0.75) (Figure 5C). In RAS, the standard length of relatively intact *C. molitorella* in the stomachs of *O. potamophilus* ranged from 0.87 to 5.99 cm. Body depth ranged from 0.16 to 1.39 cm. The relationship between SL and PSL was PSL = 0.683 × SL − 1.9816 (*n* = 10; R^2^ = 0.77) (Figure 5B). The relationship between MW and BD was BD = 0.776 × MW − 1.4639 (*n* = 10; R^2^ = 0.79) (Figure 5D). Predator size was positively associated with prey size in both culture models. Additionally, the body size of *C. molitorella* identified in the stomach contents of specimens from the RAS was larger than that of conspecifics from the RCO.

### 3.3. Body Nutritional Composition and Serum Biochemical Indices of O. potamophilus

The crude protein content of *O. potamophilus* in the RCO group was 17.41 ± 0.96%, which was significantly higher than that in the RAS group (13.89 ± 0.88%). In contrast, the contents of crude fat (RCO: 13.07 ± 1.05%, RAS: 19.51 ± 1.08%, dry-matter basis), the ALT (RCO: 35.20 ± 3.27 U/L, RAS: 141.40 ± 11.70 U/L), AST (RCO: 34.69 ± 3.53 U/L, RAS: 153.85 ± 9.41 U/L), and the GLU (RCO: 4.86 ± 0.66 mmol/L, RAS: 6.20 ± 0.59 mmol/L) were all significantly higher in the RAS group. The ash content of RCO was measured as 1.08 ± 0.23%, while that of RAS was 1.19 ± 0.11%. For moisture content, RCO recorded a value of 78.82 ± 2.23%, and RAS recorded 79.36 ± 1.56%. Statistical analysis showed that crude protein content was significantly higher in RCO than in RAS (*p* < 0.05, Figure 6A). The other four indices were significantly higher in RAS. These were crude fat, ALT, AST, and GLU (*p* < 0.05, Figure 6B–E). Ash and moisture contents showed no significant difference between the two culture models (*p* > 0.05, Figure 6F,G).

## 4. Discussion

### 4.1. Differences in Growth and Physiological Indicators of O. potamophilus Under Different Culture Models

Compared with RCO, *O. potamophilus* in RAS showed rapid growth characteristics. The final standard length of individuals cultured in the RAS was significantly greater than that of those reared in the RCO, and the final body weight in RAS was approximately 1.5 times the value observed in RCO. The power exponent for the length–weight relationship of *O. potamophilus* reared in RAS was estimated to be 3.53, which is higher than the corresponding value of 3.21 obtained from the RCO group. This difference in allometric growth was accompanied by a significantly higher Fulton’s condition factor in RAS than in RCO (*p* < 0.001), indicating that RAS fish exhibited better body condition [19,20]. Studies have shown that culture models determine fish growth primarily through three factors. These are food composition, foraging difficulty, and locomotion cost [21,22]. In RAS, the continuous supply of high-protein feed and *C. molitorella* constituted a simplified food spectrum. Fish could feed continuously without searching, freeing energy input from foraging constraints, while the gentle water current (0.1–0.3 m/s) in RAS imposed lower locomotion costs than the large-scale foraging and cruising required in RCO, resulting in energy surplus accumulation [23,24]. Excess energy was preferentially deposited in adipose tissue, resulting in a faster rate of weight gain relative to the increase in standard length [19,20]. The calculated power exponent was 3.53, which confirms the presence of typical positive allometric growth [1,25].

*O. potamophilus* cultured in RAS exhibited higher body fat content and lower protein content. Both AST and ALT levels were markedly elevated compared with those in RCO, and the GLU level was also significantly higher than that in RCO (*p* < 0.05). These results indicate that the rapid growth of *O. potamophilus* in RAS was accompanied by elevated serum transaminase activities and increased body lipid deposition, suggesting hepatic metabolic strain, although histological evidence was not collected in this study [26,27]. Excessive body fat coupled with low protein content weakens the mechanical stimulus to bones and muscles. In addition, the gentle water current in RAS (0.1–0.3 m/s) provided limited hydrodynamic resistance compared with natural flowing waters, and bones and muscles fail to receive sustained mechanical stretching, which may potentially impair the long-term locomotor capacity and commercial traits of *O. potamophilus* [15,28,29]. Therefore, the RAS should adopt integrated intervention measures, including nutritional balance regulation, feeding rhythm adjustment, and water flow stimulation. These measures can alleviate hepatic metabolic strain and reshape the tissue distribution ratio of muscle and fat, thereby achieving both rapid growth and improved physiological status.

In the RCO, fish are required to forage and cruise frequently. This sustained foraging activity promotes a healthy body condition characterized by low body fat content and high muscle protein content. Accordingly, serum biochemical indices showed stable lipid and glucose metabolism. ALT and AST activities remained at lower levels in RCO, suggesting limited hepatic lipid accumulation pressure [26,27]. It is also noteworthy that the power exponent of the length–weight relationship in the present culture model is 3.21, indicating a positive allometric growth pattern that differs from the negative allometric growth (b = 2.92) reported for rice field culture and pond culture [11]. This suggests that RCO can effectively stimulate the weight gain potential of *O. potamophilus*. RCO relies on multi-trophic integration. In this system, rice plants provide shade and reduce water temperature, crayfish consume residual feed, and *O. potamophilus* prey on weak juveniles, achieving water self-purification, disease self-suppression, and high-value products [4]. This approach represents a green and efficient ecological culture model. Follow-up interventions can include optimizing the layout of feeding platforms and moderately supplementing artificial compound feed, which can reduce energy expenditure for locomotion and further improve overall production efficiency.

### 4.2. Relationship Between Food Diversity and Growth Differences

RAS relies on a simplified, formulated feeding regime based on *C. molitorella* supplemented with fish paste and pellet feed, delivered twice daily at fixed times. The feeding window is short, and energy input is continuous, resulting in significantly higher growth parameters in RAS than in RCO. Food diversity in RCO was higher than that in RAS. It was also higher than that in rice field culture and pond culture, where crayfish was the primary food [11]. However, food resources in the RCO exhibited spatiotemporal dispersion. *C. molitorella* presented a low occurrence rate, accompanied by a high vacuity rate, and these factors were collectively associated with its slow growth [30]. Therefore, food availability may contribute more to growth performance than food diversity.

In both culture models, larger *C. molitorella* were consumed as *O. potamophilus* standard length increased. This reflects a typical predatory relationship associated with positive allometric growth [31,32,33]. This pattern matches the general strategy of carnivorous fish to reduce chasing frequency and increase dry weight per stomach content [18,34]. In RAS, *O. potamophilus* amplified the efficiency of this strategy through abundant *C. molitorella*, stable optimal temperature, and negligible foraging pressure. In addition, mean MW in RAS (1.73 ± 0.35 cm) was higher than that in RCO (1.62 ± 0.36 cm), which likely reflects the larger body size of RAS fish rather than a difference in mouthpart morphology. Moreover, the intact *C. molitorella* in stomachs also showed larger standard length and body depth in RAS than in RCO. These factors facilitated rapid growth of *O. potamophilus*. However, in RCO, uneven spatiotemporal distribution of prey increased the proportion of crayfish in the diet. Crayfish are characterized as poor swimmers, which renders them relatively easy to capture. Shrimp exoskeletons are difficult for organisms to digest and exhibit low fat content. Collectively, these factors result in decreased daily energy intake and consequently slower growth rates for predators [35]. Therefore, future culture practices should reduce crayfish stocking density and moderately supplement high-protein artificial feed. This can alleviate vacuity and slow growth in RCO caused by insufficient total food availability. These nutritional adjustments could be combined with environment-adapted culture practices, in which rearing conditions are matched to the spontaneous behavioral preferences of fish [36,37]. Such preference-based environmental design has been proposed to improve fish welfare [38,39] and may offer a direction for optimizing the physiological status of fish in both culture models, pending validation with direct behavioral and physiological indicators.

### 4.3. Implications of Culture Model Differences for Batch Production

The results of this study showed that the growth performance of *O. potamophilus* in RCO was lower than that in RAS. However, the crude protein content of *O. potamophilus* was significantly higher in RCO, and ALT, AST, and GLU remained at lower levels than those in RAS. This suggests that RCO maintained lower serum transaminase and glucose levels than RAS. Nevertheless, food resources exhibited spatiotemporal dispersion in the RCO. The frequency of occurrence of *C. molitorella* was only 46%, and the vacuity index of *O. potamophilus* in this culture model reached 38%. The unstable availability of natural feed is a core factor constraining growth potential. Accordingly, feed diversity should be maintained in mass production operations. Meanwhile, the layout of the feed platform requires optimization, and artificial compound feed should be supplemented at an appropriate level to stabilize the energy supply of the system. The growth rate can be enhanced while keeping body fat and metabolic markers within the ranges observed in RCO, thereby realizing the coordinated optimization of physiological status and production performance.

In RAS, the high-frequency feeding regime reduces foraging difficulty and locomotion cost. However, levels of AST, ALT, and GLU increased concurrently, suggesting hepatic metabolic strain. Continued long-term culture may increase individual mortality risk without corresponding economic benefits [40]. Therefore, subsequent culture may draw on the ecological characteristics and food diversity of RCO. If conditions permit, some fish can be periodically transferred to semi-natural channels. Intermittent flow regimes and gradual temperature acclimation can be implemented to simulate natural hydrological and thermal fluctuations. Maintaining healthy physiological status and restoring natural behavioral traits can preserve better body quality and improve subsequent economic benefits.

This study was conducted in two commercial culture models under actual operation. The two models differed simultaneously in rearing system, stocking density, feeding regimen, and water temperature regime (26.8 ± 2.4 °C in RCO versus 25.3 ± 1.1 °C in RAS). Therefore, the experimental design does not allow the separation of the effects of these factors, and the results reflect the overall effects of the two culture models as a whole. Any causal attribution of faster growth or physiological changes specifically to the rearing system alone should be avoided. In addition, serum biochemical analyses covered only ALT, AST, and GLU, which do not evaluate the biliary or synthetic functions of the liver. A comprehensive assessment of liver health would require additional markers such as GGT, ALP, and bilirubin, as well as histological examination. Furthermore, only the dominant food type was recorded for each stomach during stomach content analysis, which may have underestimated dietary diversity to some extent. Moreover, individual fish sampled from the same experimental unit were not fully independent, and unit-level identifiers were not retained for the sampled individuals. Although multi-point sampling was used to maximize representativeness, this hierarchical structure should be more rigorously addressed in future studies. Future studies are encouraged to establish single-factor controlled experiments to separate the independent effects of density, feed, temperature, and other factors. The number of culture replicates and the sample sizes of each indicator should also be increased, and the culture duration should be extended to evaluate long-term effects. On this basis, optimization measures such as moderate supplementary feeding in RCO and dietary diversification in RAS can be tested in validation experiments, providing a more solid theoretical basis for the coordinated optimization of the two culture models.

## 5. Conclusions

This study compared the growth, serum biochemical indices, body composition, and feeding characteristics of *O. potamophilus* reared for 92 days in two commercial culture models: recirculating aquaculture system (RAS) and rice–crayfish–*Odontobutis potamophilus* integrated culture (RCO). The experimentally demonstrated findings were as follows: Fish in RAS exhibited significantly higher final standard length, final body weight, body weight gain, relative weight gain rate, and specific growth rate than those in RCO, together with a higher Fulton’s condition factor. Fish in RAS also showed significantly higher muscle crude fat and serum ALT, AST, and GLU levels, whereas fish in RCO showed higher muscle crude protein and lower serum indices. Stomach content analysis showed that fish in RCO fed on diverse natural food items but had a high vacuity rate, whereas fish in RAS consumed almost exclusively *C. molitorella*. Because the two models differed simultaneously in rearing system, stocking density, feeding regimen, and water temperature, these differences reflect the overall effects of the two models rather than the effect of any single factor. The elevated serum transaminase activities and lipid deposition in RAS fish may indicate hepatic metabolic strain, which warrants confirmation by histological and additional biochemical analyses. Whether moderate supplementary feeding in RCO and dietary diversification, intermittent water flow, and temperature acclimation in RAS can reconcile growth and physiological status remains to be tested in controlled experiments. These findings provide a reference for optimizing the culture of *O. potamophilus* by integrating the strengths of both models.

## Figures and Tables

**Figure 1:**
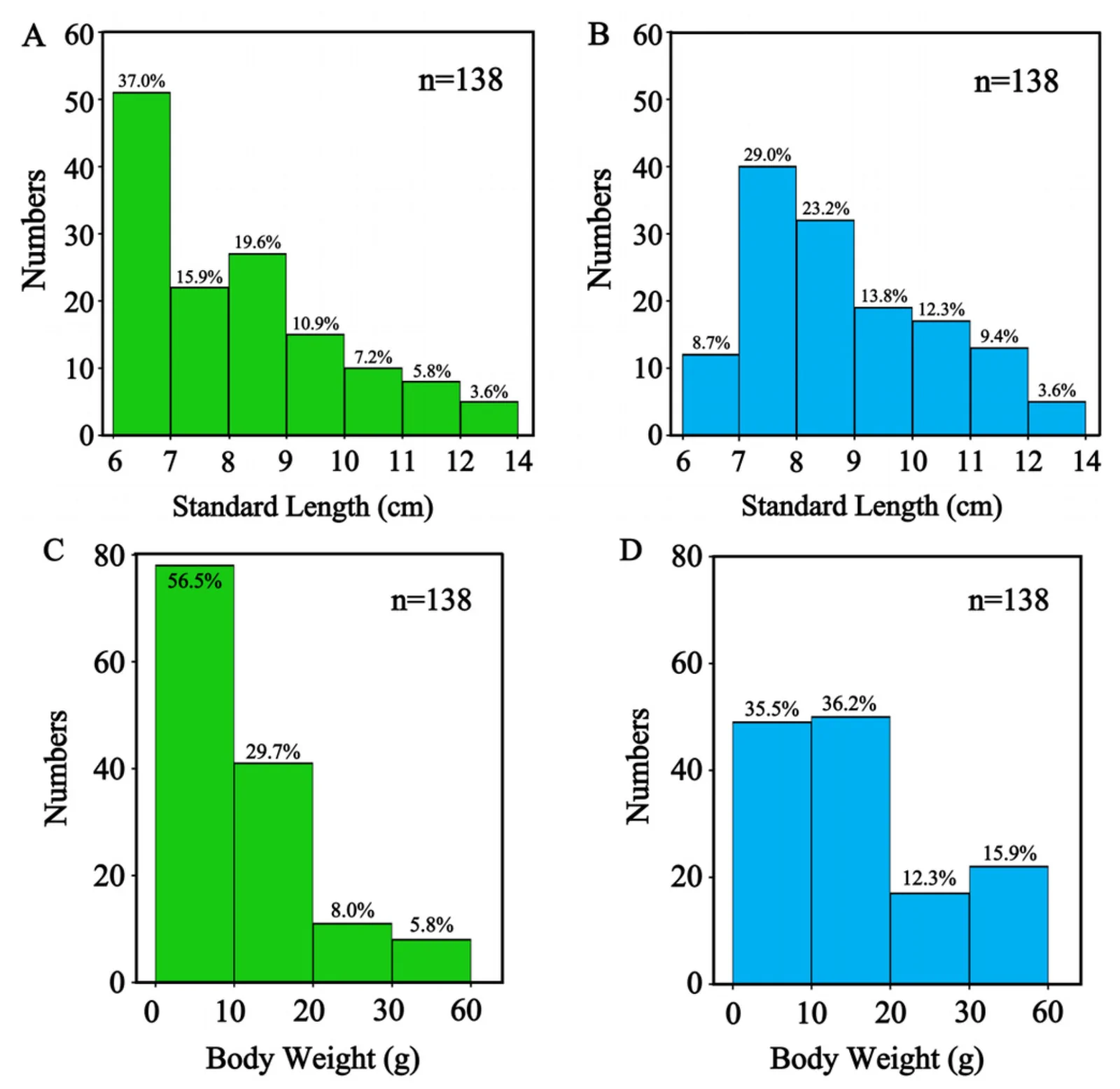
Relative frequency distribution of standard length (**A**,**B**) and body weight (**C**,**D**) of *Odontobutis potamophilus*. Panels (**A**,**C**) show data from rice–crayfish–*Odontobutis potamophilus* integrated culture. Panels (**B**,**D**) show data from the recirculating aquaculture system.

**Figure 2:**
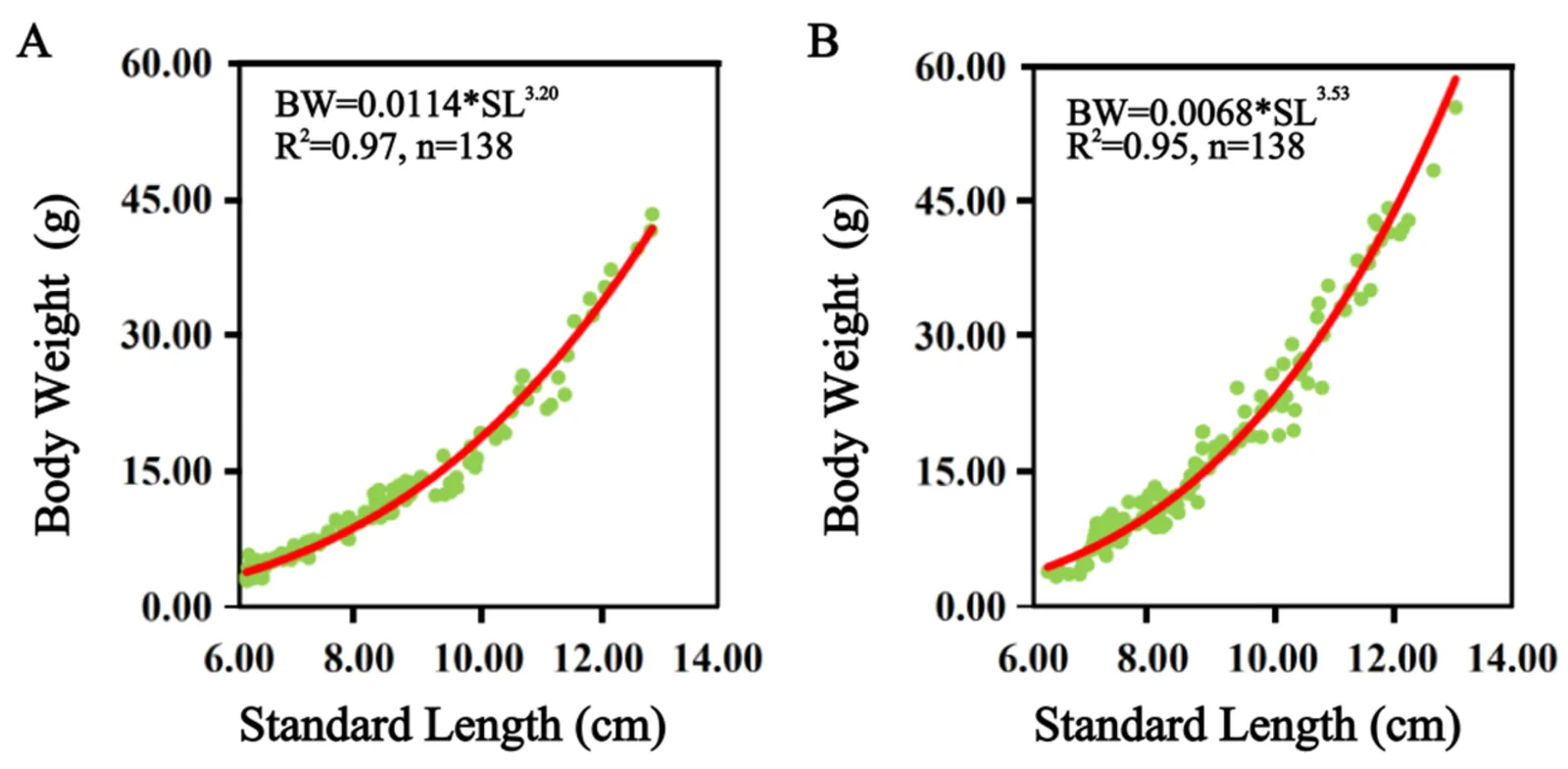
Standard length–body weight relationship of *Odontobutis potamophilus* in rice–crayfish–*Odontobutis potamophilus* integrated culture (**A**) and recirculating aquaculture system (**B**).

**Figure 3:**
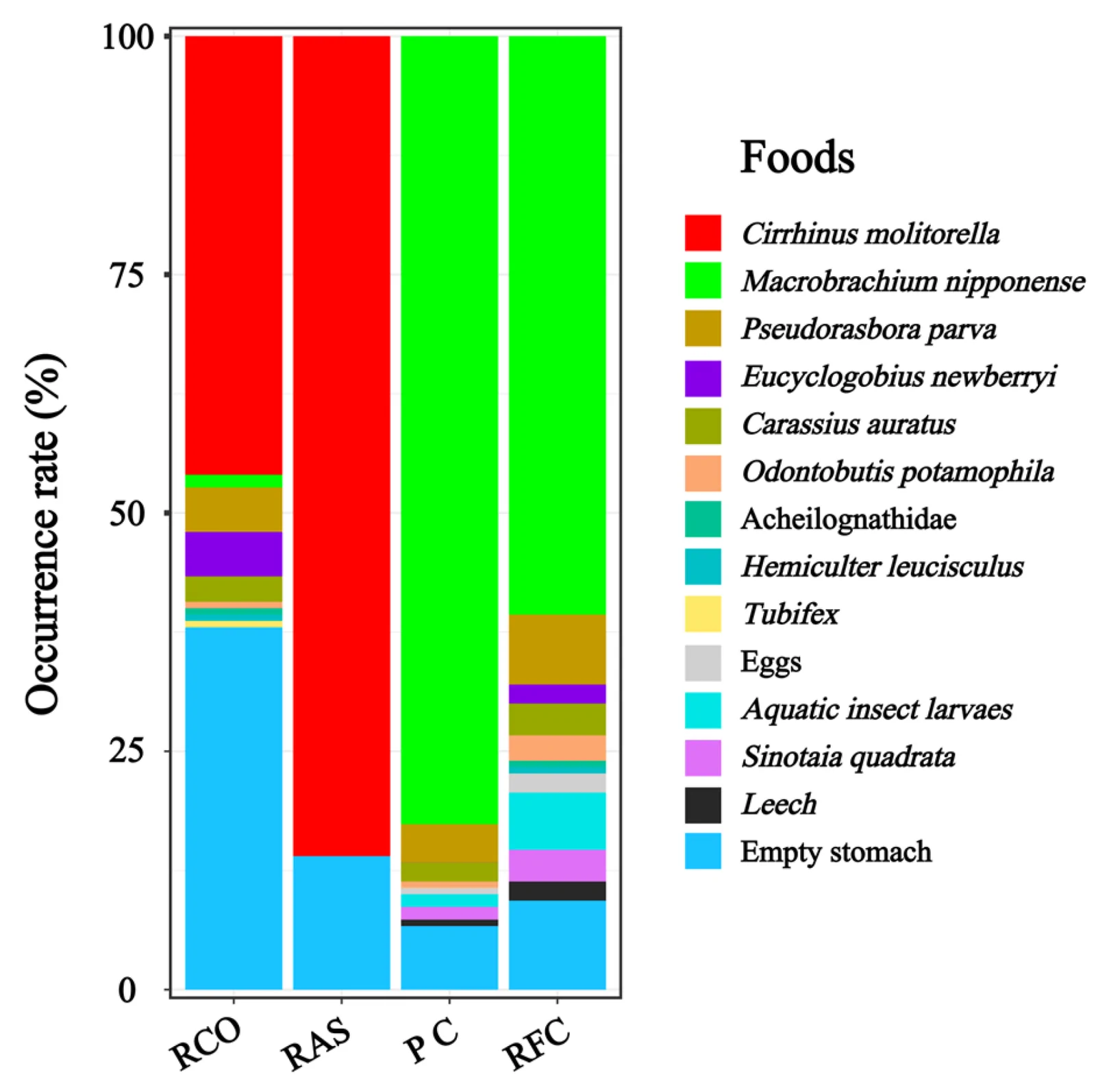
Occurrence frequency of food items recorded as the dominant stomach content of *Odontobutis potamophilus* under rice–crayfish–*Odontobutis potamophilus* integrated culture (RCO), recirculating aquaculture system (RAS), pond culture (PC), and rice field culture (RFC). Data for pond culture and rice field culture were adapted from [11] for comparative purposes.

**Figure 4:**
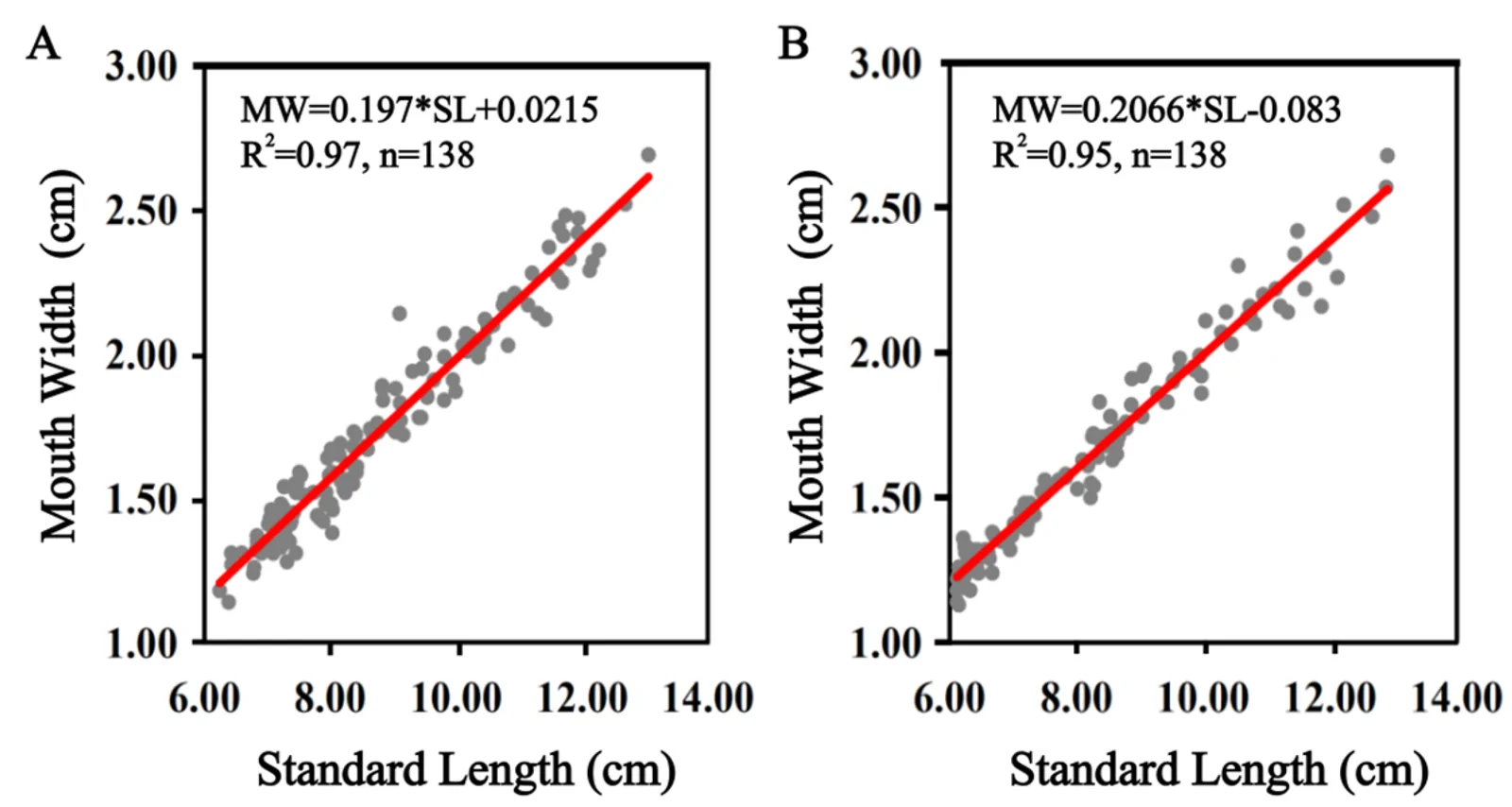
Linear relationship between standard length and mouth width of *Odontobutis potamophilus* in rice–crayfish–*Odontobutis potamophilus* integrated culture (**A**) and recirculating aquaculture system (**B**).

**Figure 5:**
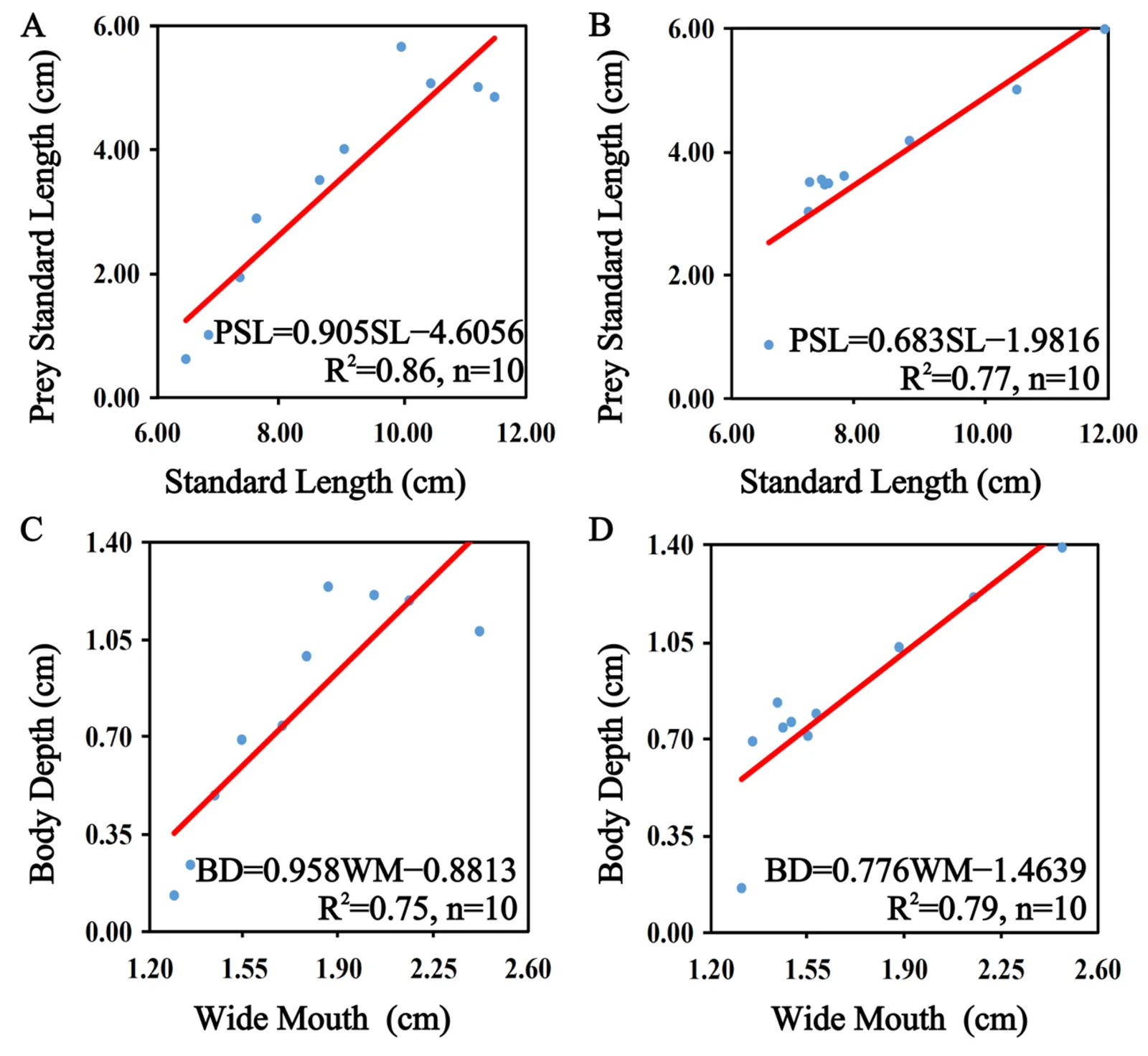
Relationship between standard length of *O. potamophilus* and prey standard length of *C. molitorella* (**A**,**B**) and relationship between mouth width of *O. potamophilus* and body depth of *C. molitorella* (**C**,**D**) under rice–crayfish–*Odontobutis potamophilus* integrated culture (**A**,**C**) and recirculating aquaculture system (**B**,**D**).

**Figure 6:**
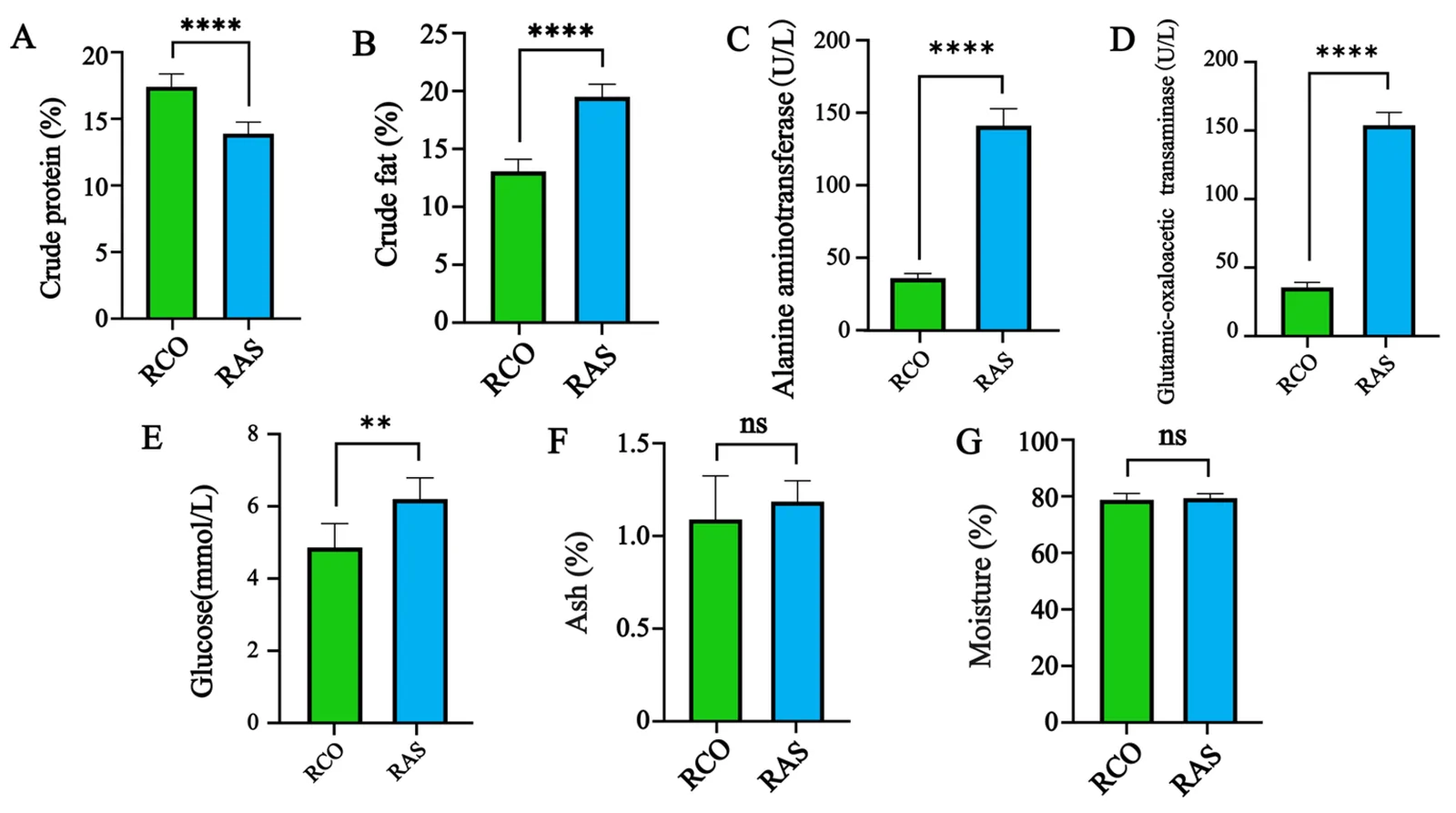
Comparison of body nutritional composition and serum biochemical indices of *Odontobutis potamophilus* between rice–crayfish–*Odontobutis potamophilus* integrated culture and recirculating aquaculture system: (**A**) crude protein; (**B**) crude fat; (**C**) alanine aminotransferase; (**D**) glutamic–oxaloacetic transaminase; (**E**) glucose; (**F**) ash; (**G**) moisture. Note: ns indicates no significant difference; ** indicates *p* < 0.01; **** indicates *p* < 0.0001. Crude fat is expressed on a dry-matter basis; the other indices are expressed on a wet-weight basis.

**Table 1:** Rearing parameters of *O. potamophilus* in rice–crayfish–*Odontobutis potamophilus* integrated culture and recirculating aquaculture system.

Culture Model	Rice–Crayfish–*Odontobutis potamophilus* Integrated Culture	Recirculating Aquaculture System
Number of culture ponds/tanks	6	6
Area (m^2^)	Each paddy field was approximately 666.7 m^2^. The ring ditch area was 200 m^2^.	Each tank was 200 m^2^.
Water depth (m)	Water depth in paddy fields was maintained at 0.1–0.25 m.	Water depth was maintained at 0.5 m.
Ring ditch (paddy field)	Ring ditch width was 2 m. Water depth was 1 m.	/
Stocking density of *Odontobutis potamophilus*	3 individuals/m^2^ (2000 individuals per paddy field, calculated relative to the total field area of 666.7 m^2^, including the ring ditch)	6 individuals/m^2^ (1200 individuals per tank, equivalent to 12 individuals/m^3^ at a water depth of 0.5 m)
Stocking size of *Odontobutis potamophilus*	0.87 ± 0.08 g/individual	0.91 ± 0.09 g/individual
Stocking density of berried crayfish	340 individuals/m^2^	/
Stocking size of berried crayfish	0.52 ± 0.13 g/individual	/
Stocking density of *Cirrhinus molitorella*	1660 individuals/m^2^	5000 individuals/m^2^

Note: In the RCO system, a shallow-water culture system, stocking density was expressed relative to the water surface area. In the RAS, density was expressed relative to both surface area and water volume.

**Table 2:** The growth performance indicators of *O. potamophilus* under rice–crayfish–*Odontobutis potamophilus* integrated culture and recirculating aquaculture system.

Culture Model	Rice–Crayfish–*Odontobutis potamophilus* Integrated Culture	Recirculating Aquaculture System
Initial standard length (ISL, cm)	1.74 ± 0.15	1.79 ± 0.18
Initial body weight (IBW, g)	0.87 ± 0.08	0.91 ± 0.09
Final standard length (FSL, cm)	8.13 ± 1.82 ^a^	8.81 ± 1.65 ^b^
Final body weight (FBW, g)	11.26 ± 8.73 ^a^	17.04 ± 11.53 ^b^
Body weight gain (BWG, g)	10.39 ± 8.73 ^a^	16.13 ± 11.53 ^b^
Relative weight gain rate (RWG, %)	1194.6 ± 1003.4 ^a^	1772.7 ± 1267.2 ^b^
Specific growth rate in wet weight (SGR_w_, %/d)	2.51 ± 0.75 ^a^	2.95 ± 0.72 ^b^

Note: Values represent the mean ± standard deviation. Different lowercase letters within the same row indicate significant differences (*p* < 0.05). Growth indicators were calculated from individual data of the sampled fish (*n* = 138 per model); the experiment lasted 92 days. As individual fish from the same experimental unit are not fully independent, these individual-level comparisons should be interpreted with caution.

**Table 3:** Number of food occurrences in the stomach of *Odontobutis potamophilus* under four different culture models.

Food Items	RCO	RAS	PC	RFC
*Cirrhinus molitorella*	69	129	0	0
*Pseudorasbora parva*	7	0	6	11
*Eucyclogobius newberryi*	7	0	0	3
*Carassius auratus*	4	0	3	5
*Macrobrachium nipponense*	2	0	124	91
*Odontobutis potamophilus*	1	0	1	4
Acheilognathidae	1	0	0	1
*Hemiculter leucisculus*	1	0	0	1
*Tubifex*	1	0	0	0
Eggs	0	0	1	3
Aquatic insect larvae	0	0	2	9
*Sinotaia quadrata*	0	0	2	5
Leech	0	0	1	3
Empty stomach	57	21	10	14

Note: Values represent the number of stomachs in which each food item was recorded as the dominant content.

## Data Availability

The original contributions presented in this study are included in the article. Further inquiries can be directed to the corresponding authors.
